# *Bacillus thuringiensis* Toxins: Functional Characterization and Mechanism of Action

**DOI:** 10.3390/toxins12120785

**Published:** 2020-12-10

**Authors:** Yolanda Bel, Juan Ferré, Patricia Hernández-Martínez

**Affiliations:** 1 Instituto de Biotecnología y Biomedicina (BIOTECMED), Universitat de València, 46100 Burjassot, Spain; 2 Department of Genetics, Universitat de València, 46100 Burjassot, Spain

*Bacillus thuringiensis* (*Bt*)-based products are the most successful microbial insecticides to date. This entomopathogenic bacterium produces different kinds of proteins whose specific toxicity has been shown against a wide range of insect orders, nematodes, mites, protozoa, and human cancer cells. Some of these proteins are accumulated in parasporal crystals during the sporulation phase (Cry and Cyt proteins), whereas other proteins are secreted in the vegetative phase of growth (Vip and Sip toxins). Currently, insecticidal proteins belonging to different groups (Cry and Vip3 proteins) are widely used to control insect pests and vectors both in formulated sprays and in transgenic crops (the so-called Bt crops). Despite the extensive use of these proteins in insect pest control, especially Cry and Vip3, their mode of action is not completely understood.

The aim of this Special Issue was to gather information that could summarize (in the form of review papers) or expand (research papers) the knowledge of the structure and function of Bt proteins, as well as shed light on their mode of action, especially regarding the insect receptors. This subject has generated great interest, and this interest has been materialized into the 18 papers published in this issue.

This Special Issue, “*Bacillus thuringiensis* Toxins: Functional Characterization and Mechanism of Action”, includes five review papers and 13 research papers. The review papers bring up to date important aspects of Bt pathogenicity, such as its interaction with the intestinal microbiota and the immune system of the insect [1]. The current knowledge about Vip proteins has also been reviewed [2], as has the contribution that the use of toxin mutants has made to the knowledge of the mode of action of the three-domain Cry proteins [3]. On the other hand, two more review papers recapitulate the information on the cytocidal activity of Bt proteins [4] or the insecticidal activity of Bt proteins against coleopteran pests [5]. All these review papers are of high value, allowing readers to stay updated on the different aspects of the Bt field described here.

The Special Issue also gathers information that could expand the knowledge of the structure and function of Bt proteins and sheds light on their mode of action, especially regarding the insect receptors. Publishing papers focusing on the steps that remain blurred within the mode of action of all Bt insecticidal proteins, including the three-domain Cry proteins, was one of the main goals. The role of receptors such as cadherin, ABCC2, and ABCA2 on the toxicity of Bt proteins in different lepidopterans has been investigated in three different papers [6,7,8]. In addition, other steps in the mode of action (that comprises protein solubilization, activation, binding, oligomerization, and pore formation) have also been addressed. Examples of these steps include the involvement of a novel trypsin protein for toxin activation in *Plutella xylostella*, discovered after studying a Cry1Ac resistant strain [9], and the promotion of oligomerization of the activated Cry1Ia with insect brush border midgut vesicles, in vitro [10]. The toxicity-promoting effect of a Bt chitin-binding protein that binds to the insect peritrophic matrix has also been studied [11]. Moreover, the Special Issue includes a paper highlighting the synergistic mosquitocidal activity of the parasporal Cry and Cyt proteins present in *B. thuringiensis* ser. *israelensis* [12], and it also includes a manuscript focused on deciphering the amino acid residues important for the interaction of Cyt2A protein with membrane lipids, a binding step necessary to exert its cytolytic action [13].

The vegetative insecticidal proteins (Vip3) secreted by *Bacillus thuringiensis* are nowadays considered as the new generation of insecticidal Bt toxins because of their different structural and molecular properties regarding the classical Bt 3-D Cry proteins. Vip3 toxins have been already introduced in Bt-crops to control lepidopteran pests. However, little is known about their mode of action. In the Special Issue, five papers analyze different aspects of its biology. They cover aspects ranging from its crystal structure [14] and structural–functional domain analyses [15] to different aspects in the mode of action, such as a study of a possible receptor (the alkaline phosphatase) in a resistant strain [16], the role of oligomerization in toxicity [17], and the study of intracellular events promoted by Vip3A intoxication in *Spodoptera frugiperda* Sf9 cells [18].

In summary, the Special Issue brings together papers of important scientific value in the field of Bt. The review and research papers included will help keep readers up to date on the topic and, at the same time, will contribute to increasing the vast knowledge of Bt and its insecticidal proteins. These studies will help to provide useful information for the development of new strategies to fight against pest insects, in the least aggressive and harmful but better environmental scenario.
